# P-1568. B-cell Depletion via Anti-CD20 Does Not Increase Recurrent Clostridioides difficile Infection Risk

**DOI:** 10.1093/ofid/ofaf695.1748

**Published:** 2026-01-11

**Authors:** Gregory R Madden, Isaura Rigo, William Petri

**Affiliations:** Division of Infectious Diseases & International Health, Charlottesville, VA; University of Virginia, Charlottesville, VA; University of Virginia, Charlottesville, VA

## Abstract

**Background:**

*Clostridioides difficile* causes a spectrum of disease ranging from asymptomatic colonization to severe gastrointestinal illness. Approximately 20-30% of patients will experience recurrent disease and the risk increases with every episode. The humoral immune response to *C. difficile* toxin B has been shown to be protective against *C. difficile* infection (CDI), with low levels of antibodies correlating with more severe disease and increased risk of recurrence. We conducted a retrospective study to evaluate the risk of recurrence in patients who were treated with anti-CD20 depleting agents. Anti-CD20 depleting agents are monoclonal antibodies that result in B-cell depletion from the peripheral blood and lymphoid tissues. We hypothesized that patients treated with anti-CD20 therapy would be at increased risk of recurrent disease due to a dysfunctional humoral immune response.

Treatment with a CD20 depleting agent had no significant effect on time to recurrent infection

**Fig. 1.**
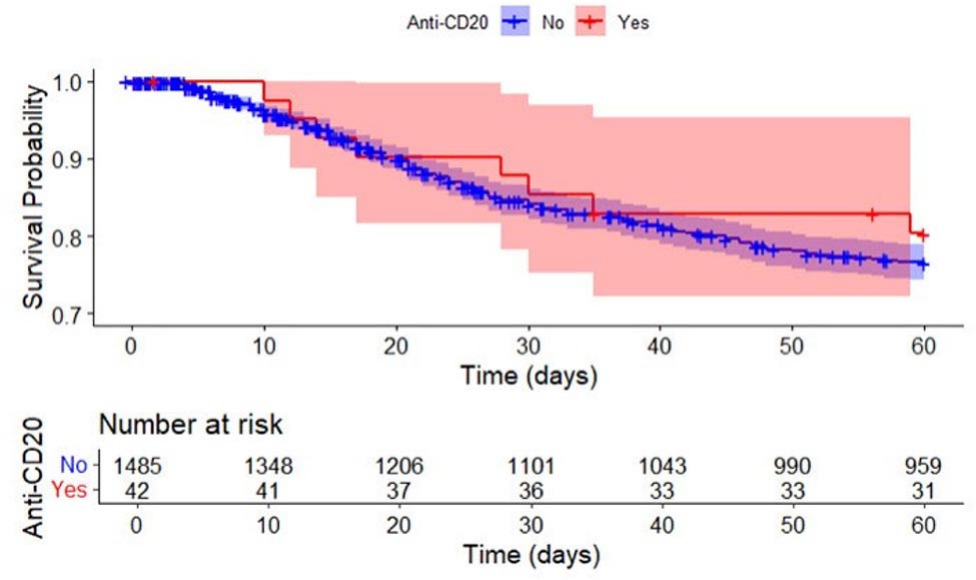
Kaplan-Meir survival curve. Effect of treatment with CD20 on recurrence-free survival. Cox regression model (adjusted HR 0.91, 95% CI (0.50–1.66, p-value = 0.75) HR = Hazard Ratio; CI = Confidence Interval.

**Methods:**

Hospitalized CDI cases were retrospectively identified at the University of Virginia Health System between January 2014- April 2021. CDI was defined as a positive PCR result followed by standard of care therapy within 48hrs of diagnosis. Recurrent CDI was defined as a positive repeat PCR test within 11-60 days post-infection or symptom relapse with re-treatment or extension of initial treatment within 60 days of index case. Children < 18 years were excluded. The Kaplan–Meier method was used to measure the recurrence-free survival.

**Results:**

There was a total of 1,519 hospitalized cases of CDI identified among 1,302 individuals. A total of 59 patients were on anti-CD20 therapy. Neither the unadjusted analysis nor multivariable cox regression (adjusting for age and recurrence number) showed a significant effect of anti-CD20 therapy given within the preceding 3 months of infection on time to subsequent recurrent infection within the 60-day follow-up period.

**Conclusion:**

There was no significant effect on time to recurrent infection after treatment with an anti-CD20 agent. It is important to note that this is a retrospective study that was performed collecting available clinical data from medical charts and we did not measure anti-toxin B antibody titers. Since anti-CD20 therapy does not target mature plasma cells they could be a potential source of antibodies from pre-existing immunity.

**Disclosures:**

William Petri, PhD, MD, TechLab: Advisor/Consultant

